# The Role of Melatonin in the Inflammatory Process in Patients with Hyperglycemia and Leishmania Infection

**DOI:** 10.3390/biom14080950

**Published:** 2024-08-06

**Authors:** Thalissa Mariana de Moraes Martins, Felipe Rubin Ferrari, Adriele Ataides de Queiroz, Letícia Damas Leão Dalcin, Danielle Cristina Honorio França, Adenilda Cristina Honório-França, Eduardo Luzía França, Danny Laura Gomes Fagundes-Triches

**Affiliations:** Institute of Biological and Health Science, Federal University of Mato Grosso, Barra do Garças 78605-091, MT, Brazil; thalissamartins02@gmail.com (T.M.d.M.M.); frferrari9@gmail.com (F.R.F.); adrieleaqueiroz@hotmail.com (A.A.d.Q.); let_damas@hotmail.com (L.D.L.D.); daniellechfranca@gmail.com (D.C.H.F.); dannylauragf@hotmail.com (D.L.G.F.-T.)

**Keywords:** inflammation, diabetes, leishmaniasis, melatonin, cytokines

## Abstract

Type 2 diabetes mellitus is a metabolic disorder that causes chronic high blood sugar levels, and diabetic patients are more susceptible to infections. American cutaneous leishmaniasis is an infectious disease caused by a parasite that affects the skin and mucous membranes, leading to one or multiple ulcerative lesions. Chronic inflammation and functional changes in various organs and systems, including the immune system, are the primary causes of both diseases. Melatonin, an essential immunomodulatory, antioxidant, and neuroprotective agent, can benefit many immunological processes and infectious diseases, including leishmaniasis. Although, limited reports are available on diabetic patients with leishmaniasis. The literature suggests that melatonin may play a promising role in inflammatory disorders. This study was designed to assess melatonin levels and inflammatory mediators in diabetic patients affected by leishmaniasis. Blood samples from 25 individuals were analyzed and divided into four groups: a control group (without any diseases), a Leishmania-positive group, patients with type 2 diabetes mellitus, and patients with a combination of both diseases. This study measured the serum levels of melatonin through ELISA, while IL-4 and TNF-α were measured using flow cytometry, and C-reactive protein was measured through turbidimetry. This study found that patients with leishmaniasis significantly increased TNF-α and decreased melatonin levels. However, the group of diabetic patients with leishmaniasis showed higher melatonin levels than the control group. These observations suggest that TNF-α may influence melatonin production in patients with American cutaneous leishmaniasis, potentially contributing to the inflammatory characteristics of both diseases.

## 1. Introduction

Type 2 diabetes mellitus is the most common form of diabetes. It is associated with two main mechanisms: resistance to insulin action in tissues such as skeletal muscle, liver, and adipose tissue, and the secretory dysfunction of pancreatic β cells. Due to the large number of people affected and the high levels of early mortality, diabetes is a public health problem [1,2,3].

One of the key factors in the diabetic state is the hyperglycemic environment. This environment triggers immunological dysfunctions, such as decreased phagocytosis and the bactericidal activity of neutrophils and macrophages [1,4,5,6]. It also affects the function and percentages of T lymphocytes. Moreover, the hyperglycemic environment changes cytokine levels, complement system proteins, and antibodies [7,8,9,10].

The change in immune response can trigger the emergence of several infectious diseases, including parasitic infections, such as leishmaniasis. Leishmaniasis is a group of neglected tropical diseases caused by a protozoan of Leishmania, order Kinetoplastida, and family Trypanomastidae, in more than 98 countries [6,11]. This disease has two main clinical forms: the tegumentary form, with cutaneous and mucosal involvement, and the visceral form, involving the spleen, liver, and bone marrow [11,12].

American cutaneous leishmaniasis is the most common form, affecting the skin and mucosa and causing single or multiple ulcerative lesions. About 95% of American cutaneous leishmaniasis cases occur in the Americas, the Mediterranean basin, the Middle East, and Central Asia. It is estimated that between 600,000 and 1 million new cases occur annually worldwide [11].

Due to its complexity, immunity against American cutaneous leishmaniasis is complicated. Several cytokines are produced, playing different roles in the resistance and susceptibility of Leishmania infection [13,14].

Resistance to American cutaneous leishmaniasis is related to the emergence of responses with the participation of CD4+ T cells with a Th1 profile, with the production of pro-inflammatory cytokines such as IL-1, IL-2, IL-12, IFN-γ, and TNF-α, which lead to macrophage activation and parasite death. While susceptibility is associated with CD8+ T cells, which have a cytolytic profile, the lysis of infected cells without killing the parasites and the increased production of cytokines and chemokines with a Th2 profile, such as IL-4, IL-5, IL-13, and TGF-β, leads to parasite replication and persistence [13,14,15,16,17].

Severe and chronic processes of inflammation are similar features in both diseases. C-reactive protein is an acute-phase protein whose levels increase rapidly within 24 to 48 h in response to various infectious or inflammatory conditions. Its supporting role in resolving infections by several microorganisms and regulating evaluation processes is increasingly recognized and comprehensive [18,19,20,21].

The current population’s lifestyle gradually shifts towards active, nocturnal, and sedentary daytime patterns, disrupting the natural circadian rhythm. This disruption is strongly linked to impaired glucose tolerance and an increased risk of type 2 diabetes [22].

Melatonin is the main hormone secreted by the pineal gland. It is found in various body fluids and has its peaks at night in all species. The hormone’s levels decrease significantly during the day, due to the inhibitory effect of visible light [23,24]. This hormone can be synthesized in other cells and tissues apart from the pineal gland, such as the retina, the gastrointestinal tract, the liver, the kidneys, the spleen, the human placenta, and immune cells, as well as through the skin [24,25]. Many of the effects of melatonin occur through its interaction with G-protein-coupled membrane-bound melatonin receptors type 1 (MT1) and type 2 (MT2), or indirectly with nuclear receptors of the RORα/RZR family, which are widely distributed throughout the body [25,26,27].

This hormone is crucial in modulating the immune system and protecting the nervous system. It promotes increased antigen presentation, phagocytic activity, and the production of NK cells, monocytes, and various cytokines. Studies have suggested that melatonin could be effective against bacterial, viral, and parasitic infections through multiple mechanisms [28,29,30]. Furthermore, studies have shown the important role of melatonin in insulin secretion and glucose regulation [31,32].

The role of melatonin in the interaction between parasites and their hosts is quite complex. While melatonin protects the host, it can also benefit the parasite during an infection. Therefore, it is important to understand whether changes in melatonin levels and other mediators, such as cytokines, occur during leishmania infection in diabetic patients. This study aims to assess melatonin levels and inflammatory mediators in diabetic patients affected by leishmaniasis.

## 2. Materials and Methods

### 2.1. Study Area

This study was conducted in Barra do Garças, Brazil, located in the Center-West Region, in Mato Grosso State, 516 km from the capital, Cuiabá, with a latitude of 15°53′24″ south. It is a municipality that presents favorable conditions for establishing and maintaining the Leishmania cycle in humans and domestic animals.

### 2.2. Inclusion and Exclusion Criteria

This study included individuals with a confirmed diagnosis of type 2 diabetes mellitus or American cutaneous leishmaniasis of both genders and ages between 18 and 65 years. Patients that used antiprotozoal drugs (Glucantime^®^) in the last 30 days and/or individuals with serious infectious diseases such as HIV/AIDS, Syphilis, and Hepatitis, were excluded from the study.

### 2.3. Subjects 

Peripheral blood samples were collected from 25 volunteers using tubes containing EDTA with a volume of 4 mL between 07:00 and 10:00 h in fasting conditions. These individuals were subdivided into four groups: those normoglycemic without Leishmaniosis infections (Control group, *n* = 7); the individuals with Leishmaniosis infections (ACL group, *n* = 6); the individuals with type 2 diabetes mellitus (T2DM group, *n* = 6); and the individuals with type 2 diabetes mellitus and with Leishmaniosis infections (T2DM-ACL group, *n* = 6).

### 2.4. Sample Processing

The blood was centrifuged for 10 min at 2000 rpm at room temperature. The plasma was reserved and stored in 2 mL microtubes in a freezer at −80 °C for later measurements of glucose, melatonin hormone, C-reactive protein, and cytokines.

### 2.5. Glucose Determination 

Blood glucose was measured in human plasma using the Glucose PP-Gold Analyze Diagnostica^®^ Kit (Belo Horizonte, Brazil), following the manufacturer’s instructions, using the enzymatic-colorimetric principle. The absorbance was read using a spectrophotometer with a wavelength of 505 nm, and the results are shown in mg/dL. 

### 2.6. Melatonin Hormone Dosage

Melatonin was extracted from plasma and quantified using an Immuno-Biological Laboratories ELISA kit (IBL, Hamburg, Germany). The kit has a lower detection limit of 1.6 pg/mL and intra-assay and inter-assay coefficients of variation (%) of 3.0–11.4 and 6.4–19.3, respectively. The melatonin extraction was conducted using the affinity chromatography method with standardized columns. The columns were placed in glass tubes and centrifuged twice with 1 mL of methanol (1 min at 200× *g*). Then, the columns were washed twice with double-distilled water (1 min at 200× *g*). Once the columns were prepared, 0.5 mL of standards, controls, and samples were applied and centrifuged for 1 min at 200× *g*. After applying the samples and standards, the columns were washed again with 1.0 mL of 10% methanol for 1 min at 500× *g*. Next, the eluate containing the hormone melatonin was extracted by adding 1.0 mL of methanol at 200× *g*. After collecting the eluate, the methanol was evaporated using a centrifugal evaporator (speed-vac). The material was then reconstituted with 0.15 mL of double-distilled water while stirring for 1 min, and immediately analyzed.

In an ELISA plate, 50 milliliters of each standard control and plasma was placed, along with 50 mL of melatonin-biotin and 50 mL of antiserum in each well. The plate was incubated at 4 °C for 20 h. After this period, the supernatant was discarded, the plate was washed three times with wash buffer, and 150 mL of the conjugated enzyme was added. After 120 min of incubation at room temperature, the plate was washed three times, and 200 mL of the substrate p-nitrophenyl phosphatase (PNPP) was added, followed by another 40 min of incubation under agitation. After this period, the reaction was stopped with 50 mL of “PNPP stop” solution. The reading was then performed using a plate spectrophotometer at 405 nm. The results were obtained using a standard curve and expressed in pg/mL.

### 2.7. C-Reactive Protein Determination 

The C-reactive protein (CRP) levels in plasma were measured using the Latex C-reactive protein Kit—Biotécnica^®^, using the plate agglutination method for qualitative and semi-quantitative protein detection with the PCR Turbilátex Kit (BioTécnica^®^, Catalog 20.015.00, Belo Horizonte, Brazil), following the turbimetric method. Samples of 5 μL of plasma and standard were placed in 1000 μL of solution (phosphate Buffer 40 mmol, sodium azide 0.95 g/L, suspension of latex particles sensitized with goat IgG anti-human C-reactive protein). The suspensions were mixed and placed at 37 °C, and the reactions were measured immediately and 120 s afterward. The reactions were read on a spectrophotometer at 540 nm, and the results were expressed in mg/L.

### 2.8. Cytokine Detection by Flow Cytometry 

The concentrations of cytokines (IL-4 and TNF-α) present in the plasma samples were evaluated using the “Cytometric Bead Array” Kit (CBA, BD Bioscience, San Jose, CA, USA). These cytokines were analyzed using flow cytometry (FACSCalibur, BD Bioscience, San Jose, CA, USA). Before analyzing the cytokines, we verified the flow cytometer settings by following the manufacturer’s instructions and using tracking beads (BD Calibrite™ 3 Beads, BD Bioscience, San Jose, CA, USA). We then used compensation beads to establish compensation settings in FACSComp™ software 4.0 (BD Biosciences, San Jose, CA). We applied the same compensation matrix to all samples to ensure high accuracy and reliability in our methodology. The data were analyzed using the FCAP Array software 3.0 (BD Bioscience, San Jose, CA, USA).

### 2.9. Statistical Analysis

The results were presented through their means and standard deviations. In addition, A D’Agostino normality test and analysis of variance (ANOVA) and the Tukey post-test were used. Pearson’s correlation test was also applied. Biostat 5.3^®^ software [Mamirauá Institute, Belém, Pará, Brazil] was used to perform the tests used in this study. The results with *p* values lower than 0.05 (*p* < 0.05) were considered significant. The protocols should be described in detail, while well-established methods can be briefly described and appropriately cited.

## 3. Results

### 3.1. The Characterization of the Population Studied

**Figure 1:**
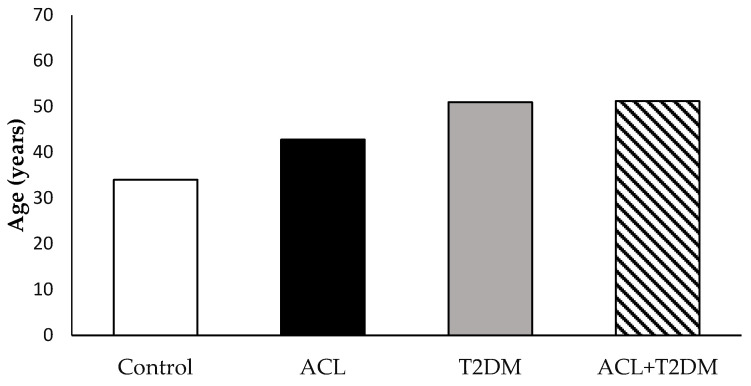
Age of the study patients. The results were evaluated using ANOVA and the Tukey test. The data were expressed as means ± SDs. *p* > 0.05.

In this study, 25 volunteers participated, with 15 being female and 10 male. The average age in the control group was 34.0 ± 12.53 years; in the ACL group, it was 42.8 ± 11.90 years; in the T2DM group, it was 51.0 ± 11.25 years; and in the group containing patients with leishmaniasis and diabetes, it was 51.2 ± 11.17 years (Figure 1). There were 12 patients diagnosed with American cutaneous leishmaniasis, 7 being women and 5 men. Among them, six lived in rural areas and six in urban areas. Six individuals had lesions in the upper limbs, four had them in the lower limbs, one had them in the abdomen, and one presented several simultaneous lesions throughout the body (mouth, nose, ear, and limbs—Table 1).

### 3.2. Plasma Blood Glucose Measurement

**Figure 2:**
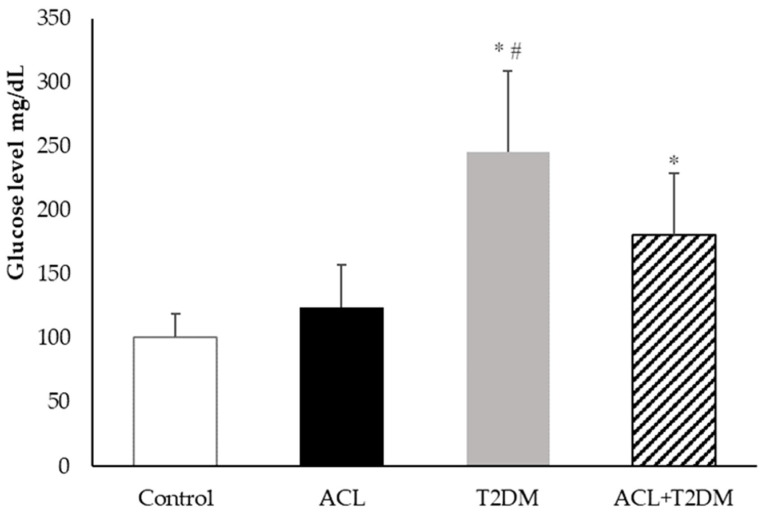
Human plasma’s glycemic concentration (mg/dL) (control *n* = 7, ACL *n* = 6, T2DM *n* = 6, ACL + T2DM *n* = 6). The results were evaluated using ANOVA and the Tukey test. The data were expressed as means ± SDs, where * indicates statistical difference (*p* < 0.01) between the experimental groups compared to the control group; and # indicates statistical difference (*p* < 0.01) when comparing the American cutaneous leishmaniasis (ACL) group with the American cutaneous leishmaniasis and type 2 diabetes mellitus (T2DM) group.

Figure 2 shows the blood glucose levels in the patient’s plasma. The glucose concentration was higher in the type 2 diabetes mellitus group (245.40 ± 63.54 mg/dL) and the American cutaneous leishmaniasis plus type 2 diabetes mellitus group (180.93 ± 48.10 mg/dL) compared to the control group (100.98 ± 18.71 mg/dL). The type 2 diabetes mellitus group had the highest glucose concentrations. There was no statistical difference in glucose levels in the American cutaneous leishmaniasis group (123.98 ± 33.70 mg/dL).

### 3.3. Melatonin Hormone Concentration

**Figure 3:**
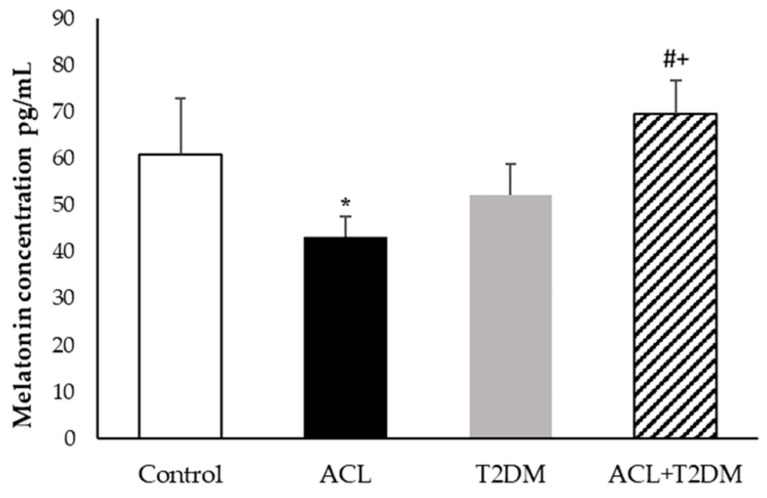
Melatonin concentration (pg/mL) in human plasma (control *n*= 7, ACL *n*= 6, T2DM *n*= 6, ACL + T2DM *n* = 6). The results were evaluated using ANOVA and the Tukey test. The data were expressed as means ± SDs, where * indicates statistical difference (*p* < 0.01) between the experimental groups compared to the control group; # indicates statistical difference (*p* < 0.01) when comparing the American cutaneous leishmaniasis (ACL) group with the American cutaneous leishmaniasis and type 2 diabetes mellitus (T2DM) group; and + indicates statistical difference (*p* < 0.01) when comparing the type 2 diabetes mellitus (T2DM) group with the American cutaneous leishmaniasis (ACL) and type 2 diabetes mellitus (T2DM) group.

The melatonin concentration was higher in individuals with diabetes and American cutaneous leishmaniasis (69.65 ± 7.03 pg/mL) and lower in individuals with leishmaniosis (43.12 ± 4.44 pg/mL) when compared to the control group (60.79 ± 12.09 pg/mL). Patients with type 2 diabetes mellitus (54.08 ± 6.68 pg/mL) showed melatonin levels similar to those of the control group and lower when compared to the values in patients with type 2 diabetes mellitus and leishmaniosis. The highest melatonin level was observed in patients with type 2 diabetes mellitus and leishmaniosis (Figure 3).

### 3.4. C-Reactive Protein Level

**Figure 4:**
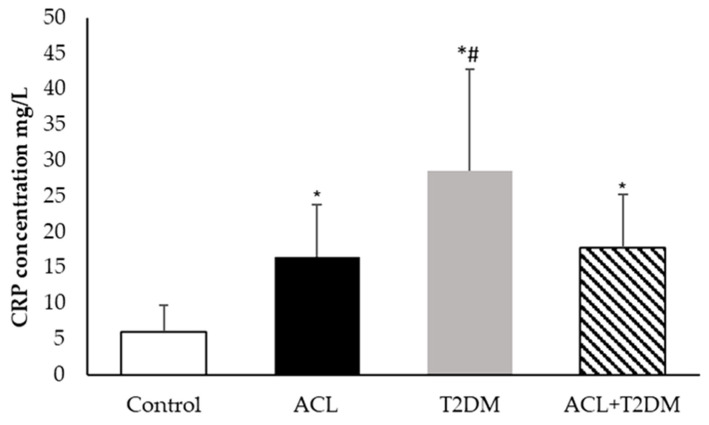
The concentration of C-reactive protein (mg/L) in human plasma (control *n* = 7, ACL *n*= 6, T2DM *n* = 6, ACL + T2DM *n* = 6). The results were evaluated using ANOVA and the Tukey test. The data were expressed as means ± SDs, where * indicates statistical difference (*p* < 0.01) between the experimental groups compared to the control group; and # indicates statistical difference (*p* < 0.01) when comparing the type 2 diabetes mellitus (T2DM) group with the American cutaneous leishmaniasis and type 2 diabetes mellitus (T2DM) group.

Figure 4 displays the C-reactive protein concentrations in different groups. Plasma C-reactive protein levels were found to be higher in patients with American cutaneous leishmaniasis (ACL −16.45 ± 7.35 mg/L), patients with type 2 diabetes mellitus (T2DM-28.55 ± 14.19 mg/L), and patients with both diseases (17.79 ± 7.41 mg/L) compared to the control group (5.94 ± 3.76 mg/L). Among these groups, patients with type 2 diabetes mellitus had the highest C-reactive protein concentrations.

### 3.5. The Concentration of IL-4 and TNF-α Cytokines

**Figure 5:**
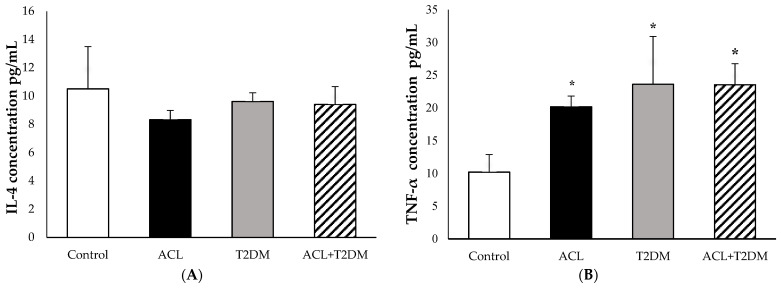
The concentration of IL-4 (**A**) and TNF-α (**B**) in (pg/mL) in human plasma (Control *n*= 7, ACL *n* = 6, T2DM *n* = 6, ACL + T2DM *n* = 6). The results were evaluated using ANOVA and the Tukey test. The data were expressed as means ± SDs, where * indicates the statistical difference (*p* < 0.01) between the experimental and control groups. ACL—American cutaneous leishmaniasis; T2DM—type 2 diabetes mellitus.

In Figure 5A and the IL-4 levels were observed in the following groups: the control group (10.51 ± 2.99 pg/mL), the American cutaneous leishmaniasis group (8.33 ± 0.65 pg/mL), the type 2 diabetes mellitus group (9.62 ± 0.62 pg/mL), and the group with both American cutaneous leishmaniasis and type 2 diabetes mellitus (9.40 ± 1.26 pg/mL). There were no statistically significant differences in IL-4 levels among the tested groups.

**Figure 6:**
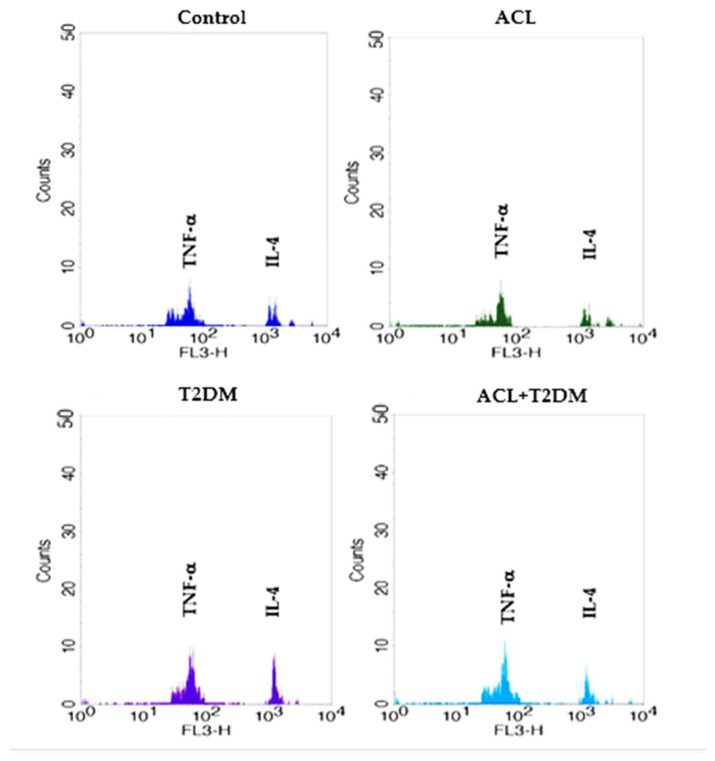
Fluorescence intensity of cytokines (IL-4, TNF-α) in serum from control, ACL T2DM, and ACL + T2DM groups. Fluorescence analyses were carried out via flow cytometry (FACSCalibur, Becton Dickinson, USA). FL3 (fluorescence in channel 3). ACL—American cutaneous leishmaniasis; T2DM—type 2 diabetes mellitus.

Figure 5B illustrates the TNF-α concentrations in plasma. Notably, patients with American cutaneous leishmaniasis (ACL—20.17 ± 1.63 pg/mL), patients with type 2 diabetes mellitus (T2DM—23.60 ± 7.29 pg/mL), and patients with both diseases (23.53 ± 3.22 pg/mL) exhibited higher TNF-α levels compared to the control group (10.20 ± 2.67 pg/mL). The fluorescence intensity of cytokines IL-4 and TNF-α can be observed in Figure 6. 

### 3.6. Correlation of Melatonin Hormone and Inflammatory Mediators

A Pearson’s correlation test was conducted to examine the relationship between melatonin levels and various factors, including patient age, glycemic indexes, and the inflammatory mediators IL-4, TNF-α, and C-reactive protein (Table 2). The analysis revealed no significant correlation between melatonin levels and patient age. However, a negative correlation was found between melatonin and glycemia in individuals with type 2 diabetes mellitus. A negative correlation was also observed between melatonin levels and C-reactive protein in the control groups (Table 2).

## 4. Discussion

Diabetes is a metabolic disease that has attracted attention due to its severity and high incidence in the global population [9]. Hyperglycemia, which results from diabetes, is associated with secondary complications that decrease patients’ quality of life and lead to premature mortality [33,34,35]. This study found that high blood glucose levels in diabetic patients associated with infection with leishmania determined alterations in melatonin levels and inflammatory mediators. 

This study found that patients with leishmania infection, without diabetes, had altered blood sugar levels. The elevated glucose levels could be due to the medications used to treat the infection, which have side effects such as nausea, vomiting, diarrhea, and increased blood sugar [36]. These side effects may explain the high blood sugar levels in these patients. The average age of the patients in this study did not exhibit statistical differences. However, it is considerable that diabetic patients, regardless of parasitic infection, were older. It is known that aging reflects progressive changes in several important physiological systems, including the immune system, continually remodeled throughout life, a process known as immunosenescence. Many age-related pathophysiological conditions, such as increased neoplasms, osteoporosis, autoimmune diseases, metabolic diseases, and increased susceptibility to infections, are directly associated with immunosenescence [37]. Melatonin levels gradually decrease throughout life and may be related to reduced sleep effectiveness, often associated with advancing age and the decline of circadian rhythms [38]. 

The literature indicates that melatonin levels can vary significantly throughout the day [39,40]. These variations are probably linked to the characteristics of the population, the type of work, and the existing disease conditions. Melatonin, a key marker of the body’s circadian rhythm, can fluctuate due to seasonal changes and an individual’s lifestyle [41,42].

Melatonin is the pineal gland’s main hormone, and changes in the immune–pineal axis have been associated with host–parasite interactions. The effects of this hormone on the body depend on several factors, including concentration, circadian rhythm, receptor affinity, the presence of infections, the flexibility of immune cells, and metabolic changes, such as diabetes [7,43,44]. This research examined daytime melatonin levels and found that hyperglycemic patients infected with *Leishmania* had increased hormone levels in their blood.

It has been shown that melatonin can directly affect parasitic infections, acting on protozoan parasites and/or the host’s immune response. Melatonin could play a crucial role in combating leishmaniasis. Elmahallawy et al. [45] found that exogenous melatonin treatment reduced the number of *Leishmania infantum* promastigotes. Laranjeira-Silva et al. [46] showed that *Leishmania amazonensis* infection did not interrupt melatonin production in the pineal gland in hamsters. This study observed lower melatonin levels in patients with American cutaneous leishmaniasis, suggesting that *Leishmania* infection may interfere with the melatonin levels circulating in parasitized individuals’ blood.

Melatonin is involved in several physiological and oxidative processes, has an anti-inflammatory effect, and regulates human glucose [7]. This hormone has been found to have a functional connection with the immune system, strongly stimulating immune cells in vivo, indicating its potential use as a therapeutic agent to enhance immune responses [47,48,49,50,51,52,53,54,55]. 

However, the hyperglycemic environment, combined with exacerbated and systemic inflammation, increases the susceptibility of diabetic individuals to infectious diseases [56,57]. Studies have found that diabetics are more susceptible to infection by *L. braziliensis* [44]. Additionally, high levels of the lipid mediator LTB4 in hyperglycemic patients result in an ineffective response against leishmania, leading to chronic inflammatory responses and impaired healing processes [58]. The elevated melatonin level in diabetic patients with leishmania infections suggests a possible mechanism for controlling systemic inflammation caused by the combination of hyperglycemia and parasite infections.

Studies have shown a positive regulation of the gene expression of pro-inflammatory cytokines (IL-1β, TNF-α, and IL-6) in human macrophages after infection with *L. amazonensis* and *L. major*. On the other hand, it was demonstrated that MLT could inhibit the production of these cytokines mediated by LPS-TLR4 signaling [59,60]. Our results showed a significant increase in TNF-α and a reduction in MLT levels in patients with American cutaneous leishmania, corroborating the data mentioned above and reinforcing the clinical data that showed the suppression of melatonin in patients with high levels of circulating TNF-α in various diseases [22,61,62]. This study measured C-reactive protein, IL-4, and TNF-α levels to determine the inflammatory status of patients who showed elevated levels of C-reactive protein and TNF-α in diabetic patients with or without parasite infections.

Melatonin has been found to inhibit the production of pro-inflammatory cytokines, suggesting that this hormone might help reduce both acute and chronic inflammation. Studies have shown that melatonin reduced inflammation in the livers of mice prone to accelerated senescence (SAMP8), resulting in increased mRNA and protein expression levels of TNF-α and IL-1β, as well as an increase in the expression level of IL-10 [63]. Other research indicates that melatonin production might be suppressed if the pineal gland becomes a target for the immune system due to pathological conditions associated with TNF-α. However, the data from this study did not show a correlation between melatonin and TNF-α levels in the blood of patients with two concomitant diseases.

The immune-inflammatory mediators, such as TNF-α, IL-1, and IL-6, can lead to insulin resistance due to oxidative stress from high glucose intake, reduce insulin sensitivity, and produce high levels of these mediators in the bloodstream. They also play a role in producing C-reactive protein, an important marker of inflammation related to tissue damage, further contributing to insulin resistance [64,65]. This study confirms these findings by showing that diabetic patients had higher TNF-α and C-reactive protein levels than the control group, indicating chronic inflammation. This state of inflammation can hinder tissue repair and make individuals more susceptible to infections. Previous research has connected elevated levels of pro-inflammatory cytokines, particularly TNF-α, to more severe tissue damage at infection sites, resulting in severe ulcers and mucosal damage [65,66]. Similar findings were observed in diabetic patients, who exhibited characteristic ulcers and, in some cases, recurring lesions. Furthermore, one diabetic patient with parasitic infections had lesions that extended to the mucosal region, confirming these earlier observations.

It is interesting to note that melatonin levels have an inverse correlation with glycemia in diabetic patients, supporting the findings of Peschke et al. [67]. Their research suggested an interaction between insulin and melatonin in patients with diabetes. They found that high glucose and insulin levels are linked to lower levels of MLT [68,69,70]. Additionally, it has been established that reactive oxygen and nitric oxide species play a role in the destruction of pancreatic islets and the development of diabetes. Melatonin helps to improve diabetes-related disorders by reducing oxidative stress and stimulating cellular antioxidant systems, highlighting that diabetic patients with reduced levels of melatonin, as shown in this research, have a significant deficit in the immune response [71,72,73].

Based on the data, there is evidence of the significant role of melatonin in regulating the immune system and its impact on infectious diseases. The importance of inflammatory mediators in the immune-related aspects of infectious and non-infectious diseases is evident. Additionally, melatonin is being extensively studied as a potential treatment for improving the quality of life in individuals affected by metabolic and parasitic diseases. However, there is still a need to understand the essential role of melatonin synthesis in an appropriate inflammatory response and to investigate whether the daily melatonin rhythm can be restored in associated diseases. Further research is needed to study the role of melatonin and the immune–pineal axis in balancing the innate and adaptive immune responses for better defense against invading pathogens.

This study’s data were evaluated during a specific collection period, which is a limitation. It is important to continue investigations focusing on other hormones and cytokines that may be involved in leishmaniosis infections in diabetic patients.

## 5. Conclusions

According to the results, TNF-α can impact melatonin production in individuals with American cutaneous leishmaniasis. This influence could potentially contribute to the inflammatory features associated with both conditions. Furthermore, research indicates that diabetic patients with American cutaneous leishmaniasis experience increased melatonin production, suggesting that this may be a compensatory mechanism for controlling these two comorbidities.

## Figures and Tables

**Table 1:** Characterization of the population studied.

	Participants (*n* = 25)
Characteristics	*n*
Gender	
Male	10
Female	15
Locality	
Rural	7
Urban	18
Injury sites (ACL)	
Upper limbs	4
Lower limbs	6
Others	2

Note: ACL—American cutaneous leishmaniasis.

**Table 2:** Correlation between melatonin levels and patients’ age, glycemic indexes, or inflammatory mediators (CRP, IL-4, and TNF-α).

	Control		ACL		T2DM		ACL + T2DM	
	Rs	*p*	Rs	*p*	Rs	*p*	Rs	*p*
Melatonin/Age	−0.470	0.423	0.090	0.880	−0.665	0.220	−0.397	0.507
Melatonin/glucose	0.083	0.894	0.876	0.051	−0.962	0.008 *	−0.814	0.093
Melatonin/CRP	−0.914	0.029 *	0.278	0.650	0.583	0.349	0.155	0.802
Melatonin/IL-4	0.126	0.873	0.549	0.450	−0.631	0.368	0.343	0.657
Melatonin/TNF-α	0.443	0.556	0.681	0.318	−0.180	0.819	−0.080	0.920

Note: * Intragroup correlation. Rs = Pearson linear coefficient. CRP stands for C-reactive protein, ACL for American cutaneous leishmaniasis, and T2DM for type 2 diabetes mellitus.

## Data Availability

The authors will make the data supporting this study’s interpretations available if requested.
